# Publishers’ Notice

**Published:** 1883-01

**Authors:** 


### PUBLISHERS' NOTICE.

Having assumed the responsibility of publishing the Independent Practitioner, it is our object to make the journal first-class in every particular.

Dr. B. M. Wilkerson, the founder and editor of this journal, in his valedictory, which was published in the December number, expresses his regrets at being compelled to retire from the editorial chair on account of personal matters which imperatively demand every moment of his time.

In his graceful yet "lingering adieu" he extends his sincere thanks to all those who have in any way rendered service to the journal, and requests that they continue to support it under its new management. He says of his successors: "With their ability and energy Vol. IV. cannot fail to surpass in merit and attractive features all its predecessors. Leigh H. Hunt, M. D., of New York City, has undertaken full charge of the medical department. Dr. Hunt, although a young man, has accomplished more than many older members of the profession who claim to have [illegible] talent. He graduated with distinction at the New York College—taking the degree of B. Sc., carrying off the chief honors and medals of his class. He subsequently took the degree of M. D. at the University of the City of New York, Medical Department, together with the first prize for general proficiency, as well as prizes for Chemistry and Physiology. Four months later he was appointed Instructor in the Pathological Laboratory of that institution, a position which he still occupies with credit to himself and satisfaction to the authorities. He has recently translated Charcot's work on old age, which has proved highly acceptable to the profession.

"W. C. Barrett, M. D., D. D. S., of Buffalo, N. Y., has accepted entire charge of the dental department. He is well and favorably known to the dental literary world as a sound and independent thinker, a bold writer and an eminent dental practitioner. Those of our readers practicing the specialty of dentistry are assured that his ability and energy will be fully employed in their service. Physicians will also be interested and edified by reading his selections and original productions."

We have reduced the price of this journal from $3 to $2.50, post-free to all parts of the United States and Canada. Postage to all other countries, however, must be added on the following scale, viz., for those within the postal union, 36 cents; for the West Indies, 72 cents (excepting the Bahamas and other islands in the postal union); for Australia, 72 cents (except New South Wales, Queensland, New Zealand and Victoria, for which the rate is 96 cents); for Bolivia and Costa Rica, $2.40. These extra rates, it is to be observed, are in each case *per annum.*

Profiting by the valuable hints derived from Dr. Wilkerson's experience, added to our own exceptional facilities and further aided as we shall be by such talent on the editorial staff and among the contributors, we are resolved to produce a journal which shall satisfy the subscribers as well as redound to the credit of the

COLUMBIA PUBLISHING COMPANY.

